# Human YRNA 4 (HY4) plasma levels are a prognostic indicator of SARS-CoV-2 infection clinical severity

**DOI:** 10.17912/micropub.biology.000925

**Published:** 2023-08-18

**Authors:** Nathaniel S. Olliff, Miles A. Hunt, Sunita S. Paudel, Kevin N. Nguyen, Haley A. Delcher, Jeffrey D. DeMeis, Justin T. Roberts, Brian W. Fouty, Jonathon P. Audia, Jin H. Kim, Glen M. Borchert

**Affiliations:** 1 Pharmacology, University of South Alabama, Mobile, Alabama, United States; 2 Biology, Howard University, Washington, Washington, D.C., United States; 3 Internal Medicine, University of South Alabama, Mobile, Alabama, United States; 4 Microbiology & Immunology, University of South Alabama, Mobile, Alabama, United States

## Abstract

SARS-CoV-2 infection can result in a range of outcomes from asymptomatic/mild disease to severe COVID-19/fatality. In this study, we investigated the differential expression of small noncoding RNAs (sncRNAs) between patient cohorts defined by disease severity. We collected plasma samples, stratified these based on clinical outcomes, and sequenced their circulating sncRNAs. Excitingly, we found YRNA HY4 displays significant differential expression (p=0.025) between patients experiencing mild and severe disease. In agreement with recent reports identifying plasma YRNAs as indicators of influenza infection severity, our results strongly suggest that circulating HY4 levels represent a powerful prognostic indicator of likely SARS-CoV-2 patient infection outcome.

**Figure 1. HY4 is significantly differentially expressed between SARS-CoV-2 patients experiencing only mild symptoms and those suffering from severe COVID-19:**
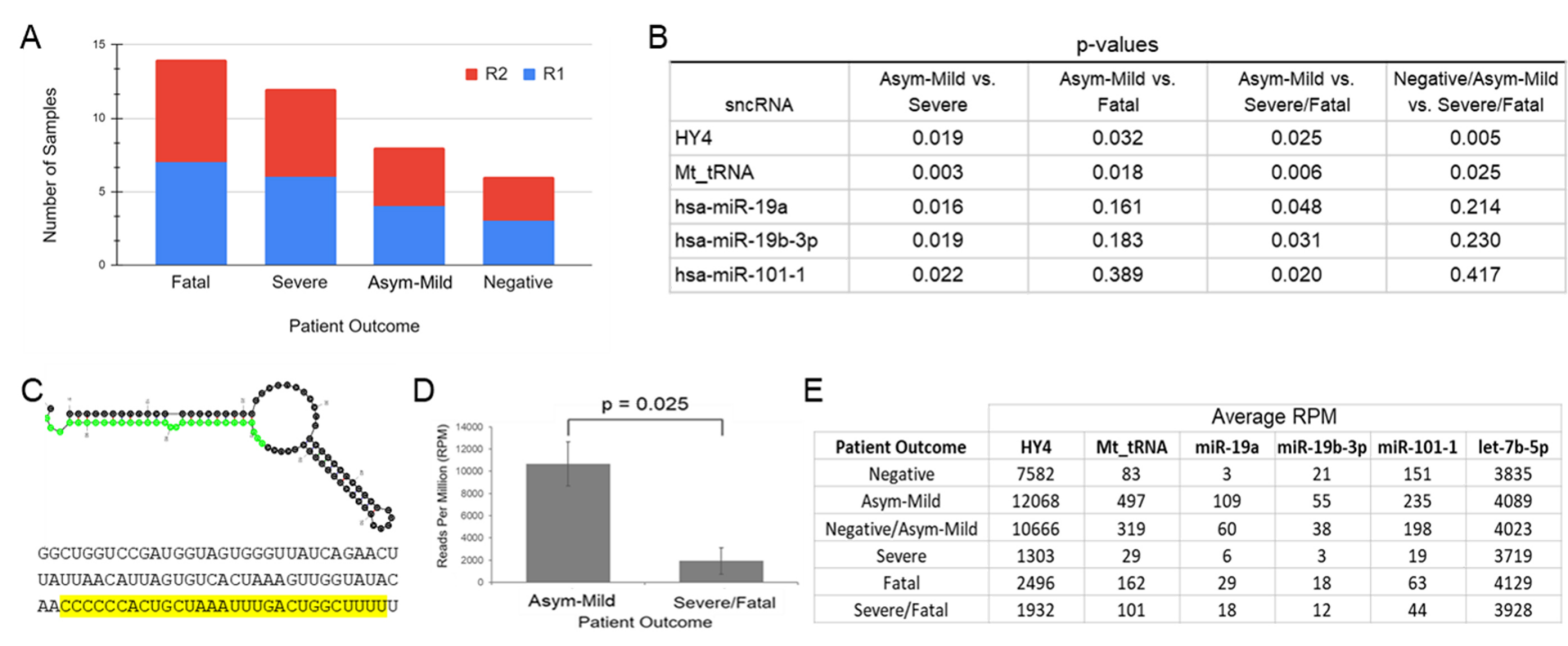
**(A)** Number of plasma sample paired-end RNA sequencing from each outcome group. Each patient sample was subjected to 5’-3’ forward-read (R1) and 3’-5’ reverse-read (R2) RNA sequencing. **(B)** Summary of sncRNAs that were found to be differentially expressed according to disease outcome. P-values are reported for each sncRNA in comparisons of outcome groups. P-values of less than 0.05 were considered significant. HY4, ENSG00000252316. Mt_tRNA, ENSG00000210176. MicroRNA annotations correspond to miRBase id. **(C)** Structure and sequence of HY4. The sequence of Ys-HY4-3p, a small RNA derived from HY4, is highlighted. **(D)** Differential expression of HY4 between patients who recovered from mild SARS-CoV-2 infection (12,068 average reads per million [RPM]) and the combined average RPM of patients who suffered from severe infection and those who died from it (1,932 average RPM). **(E)** Average expression (in RPM) of six distinct sncRNAs in each SARS-CoV-2 outcome cohort. Human miRNA Let-7b-5p was included as an example of a consistently expressed sncRNA.

## Description

Small noncoding RNAs (sncRNAs) such as microRNAs (miRNAs), small nuclear RNAs (snRNAs), small nucleolar RNAs (snoRNAs), tRNA fragments (tRFs), YRNAs, and Piwi-interacting RNAs (piRNAs) play crucial roles in the regulation of gene expression during cellular processes. SncRNAs circulate in human serum with remarkable stability, making them ideal candidates for development into non-invasive biomarkers for diagnostic, prognostic, and therapeutic decision-making purposes (Bidarra et al. 2019) . MiRNAs are the best studied sncRNAs in terms of their potential to serve as biomarkers although other sncRNAs have recently also shown promise as potential prognostic tools. Of note, host-encoded miRNAs are known to modulate antiviral defense during viral infection (Barbu et al. 2020) , and cataloging differences in circulating human miRNA levels has been reported to significantly improve the early prediction of severe disease in COVID-19 patients (de Gonzalo-Calvo et al. 2023) . That said, whether other types of sncRNAs might similarly represent valuable biomarkers for predicting SC2 patient outcomes has not been examined. As such, in this work, we asked if individual differences in levels of other types of sncRNAs found in patient plasma samples might also correlate with SC2 infection severity and outcome.

Given the global impact of the SARS-CoV-2 virus in recent years, in this study, we explored the potential of sncRNAs to serve as biomarkers for predicting the severity of SARS-CoV-2 infection in patients. To achieve this we collected plasma samples from 20 patients, stratified them based on the following clinical outcomes: SARS-CoV-2 negative (n=3), patients exhibiting only mild symptoms (n=4), patients with severe COVID (n=6), and COVID fatalities (n=7), and sequenced their circulating small RNAs via ncRNA-Seq ( **Figure 1A** ). Significant differential expressions of five sncRNAs were found ( **Figure 1B** ) although only H uman Y RNA 4 (HY4) was consistently expressed at >100 reads per million (RPM). Notably, HY4 ( **Figure 1C** ) exhibited significant differential expression when sequencing data from patients who recovered from mild SARS-CoV-2 infection (12,068 average RPM) were compared to those who suffered from severe disease (1,303 RPM) (p = 0.019), those who died from SARS-CoV-2 infection (2,496 RPM) (p = 0.032), and the combined average RPM of those who suffered from severe infection / suffered fatalities (1,932 RPM) (p = 0.025) ( **Figure 1D,E** ). Furthermore, there was significant differential expression between the combined data of patients who were negative for SARS-CoV-2 and those who recovered from mild disease (10,666 RPM) compared to those who suffered from severe disease or who died from COVID-19 (p = 0.005).

In this work, HY4 was identified as being significantly differentially expressed between patients experiencing only mild SARS-CoV-2 infection and those suffering from severe disease. Interestingly, human microRNAs miR-19a, miR-19b, and miR-101 were also identified as being significantly differentially expressed between these patient cohorts and each of these have been recently reported as correlating with SARS-CoV-2 infection severity (Hoque et al. 2022) ( **Figure 1B,E** ). That said, we found each of these miRNAs were present at considerably lower levels than HY4 in human patient plasma.

YRNAs, typically 83-112 nucleotides long, belong to a class of small noncoding RNAs that participate in essential cellular processes including DNA replication, RNA stability, and stress responses. YRNAs are particularly associated with autoimmune conditions, having been initially identified as components of the RoRNP (Ro60 RNA-binding protein ribonucleoprotein) complex observed in the serum of individuals with systemic lupus erythematosus (SLE) and Sjögren's syndrome. Notably, recent research has described roles for circulating YRNAs in inhibiting influenza replication (Liu et al. 2019) , as well as in RSV (Palsson et al. 2022), HIV-1 (Vabret et al. 2022) , and HPV (Guglas et al. 2023) viral infections, sparking interest in understanding the response of YRNAs to viruses. Furthermore, recent reports describing the potential for YRNA plasma levels to serve as powerful new biomarkers for inflammatory diseases (Driedonks et al. 2020), and perhaps most of note, for plasma levels of a YRNA fragment (originally misannotated as human miR-1975) to serve as an prognostic indicator for influenza infection clinical severity, well agree with our finding that circulating HY4 levels represent a potentially powerful prognostic indicator for SARS-CoV-2 infection outcome in patients (Liu et al. 2020) . Finally, in addition to confirming the correlations reported here in additional patient cohorts, we suggest future work should also explore the potential for a mechanistic role for HY4 in host antiviral defense as we find HY4 expression much lower in patients who suffered from severe disease.

In conclusion, this study is the first to indicate that human YRNA 4 (HY4) displays significantly differential expression in patients infected with SARS-CoV-2 based on disease outcome. That said, describing specific differences between patient sncRNA profiles correlating with disease severity and outcome has the potential to provide powerful new prognostic biomarkers for assessing infected patient prognosis. In short, we suggest relevant sncRNA levels could easily be determined in parallel with initial qRT-PCR-based viral testing (as for SARS-CoV-2) and indicate not only if a patient is positive, but also their likelihood of developing severe disease and therefore appropriate initial therapeutic and clinical measures.

## Methods

Sample Collection and Small RNA Seq: Patient classifications were defined as: Negative – patients testing negative for SARS-CoV-2 and, to their knowledge, not having been previously infected; Asymptomatic/Mild – patients testing positive for SARS-CoV-2 but recovering without hospitalization; Severe – patients testing positive for SARS-CoV-2, diagnosed with COVID-19, and admitted into the ICU; and Fatal – COVID-19 diagnosis followed by ICU mortality. Samples from severe COVID and COVID fatalities were obtained from consenting patients in the USA Health University Hospital and were collected at hospital entry before treatment with immunotherapy for IL6 (e.g. Tocilizumab), interferon beta, corticoids or ribavirin. Samples from asymptomatic patients and/or patients experiencing only mild symptoms were drawn within the first week of a positive SARS-CoV-2 test from volunteers at the Frederick P. Whiddon College of Medicine. The study also included negative cases consisting of individuals who visited the vaccination center solely for vaccination purposes. Small RNAs were isolated from patient plasma samples with the mirVana™ miRNA Isolation Kit (Invitrogen #AM1560) and then used to prepare sequencing libraries with the Small RNA-Seq Library Prep Kit (Lexogen) as in (Roberts et al. 2018) (Chen et al. 2018) (Devadoss et al. 2021) . The libraries were sequenced on a NovaSEQ 6000 (Illumina) to generate 2x150bp paired end runs consisting of ~10 million raw reads per sample.

SPAR: To comprehensively profile the small RNA signatures associated with different SARS-CoV-2 clinical outcomes, the resulting reads were analyzed using SPAR (Small RNA-seq Portal for Analysis of sequencing expeRiments) (Kuska et al. 2018). SPAR is a web-based platform specifically designed to facilitate the analysis, annotation, and visualization of sncRNA sequencing data. Briefly, the pipeline performs adapter trimming and read alignment to the human reference genome (GRCh38) and then characterizes the sncRNAs according to class (i.e., miRNA, piRNA, tRNA, etc.). These results were used to identify significant differences in sncRNA expression associated with patient outcomes.

BLAST: In addition to SPAR, we also conducted our own expression analysis using BLAST+ (Basic Local Alignment Search Tool). This workflow consisted of identifying alignments between the RNA-seq reads and an annotated list of known sncRNAs and then normalizing the alignments to the total number of BLAST hits for their corresponding patient samples, and a reads per million (RPM) value was calculated for each sncRNA in each sample.

Statistical Analysis: The mean RPM value for each sncRNA of interest, including HY4, was calculated for each patient sample, including all forward (R1) and reverse (R2) reads following rRNA subtraction. Two-tailed, heteroscedastic Student’s t-tests were then performed to compare sncRNA expression between different clinical outcomes as in (Ma et al. 2020) (Li et al. 2020) (Williams et al. 2021) . The outcome groups compared using t-tests were as follows: recovered from mild cases versus severe cases, recovered from mild cases versus fatal cases, recovered from mild cases versus the combined RPM values of both the severe and fatal cases, and the combined RPM values for both SARS-CoV-2-negative cases and patient who recovered from mild infection versus the combined RPM values of severe and fatal cases.
